# Gadolinium Deposition in Brain: Current Scientific Evidence and Future Perspectives

**DOI:** 10.3389/fnmol.2018.00335

**Published:** 2018-09-20

**Authors:** Bang J. Guo, Zhen L. Yang, Long J. Zhang

**Affiliations:** ^1^Department of Medical Imaging, Jinling Hospital, Nanjing Clinical School, Southern Medical University, Nanjing, China; ^2^Department of Medical Imaging, Jinling Hospital, Medical School of Nanjing University, Nanjing, China

**Keywords:** gadolinium-based contrast agents, magnetic resonance imaging, T1 hyperintensity, gadolinium deposition, brain

## Abstract

In the past 4 years, many publications described a concentration-dependent deposition of gadolinium in the brain both in adults and children, seen as high signal intensities in the globus pallidus and dentate nucleus on unenhanced T1-weighted images. Postmortem human or animal studies have validated gadolinium deposition in these T1-hyperintensity areas, raising new concerns on the safety of gadolinium-based contrast agents (GBCAs). Residual gadolinium is deposited not only in brain, but also in extracranial tissues such as liver, skin, and bone. This review summarizes the current evidence on gadolinium deposition in the human and animal bodies, evaluates the effects of different types of GBCAs on the gadolinium deposition, introduces the possible entrance or clearance mechanism of the gadolinium and potential side effects that may be related to the gadolinium deposition on human or animals, and puts forward some suggestions for further research.

## Introduction

Gadolinium-based contrast agents are widely used as CE-MRI agents for diagnosing or monitoring disease progress. In each year, over 30 million doses of GBCAs are consumed worldwide, and more than 300 million doses have been administrated since their introduction (Gulani et al., 2017). Clinically available GBCAs are all bonded by a ligand when they are used as an MRI contrast agent because free gadolinium is highly toxic (Rogosnitzky and Branch, 2016). Until 2006, all GBCAs were considered extremely safe. In 2006, a report (Marckmann et al., 2006) stated that some GBCAs may lead to NSF in patients with renal failure. However, when performing careful evaluation of the renal glomerular filtration rate before CE-MRI, new NSF cases have not been reported. Since 2013, the safety of GBCAs has attracted broad attentions over the world. A research group from Japan reported (Kanda et al., 2014) that signal intensity in the GP and DN on unenhanced T1 weighted imaging (T1WI) may be a result of the previous GBCAs administrations. This phenomenon leads to reconsideration of the safety of GBCAs. Following this report, many studies (Errante et al., 2014; McDonald et al., 2015; Miller et al., 2015; McDonald et al., 2017) focused on the potential risks of gadolinium retention in the human brain.

In this review, we summarize the current evidence on gadolinium deposition in the human and animal bodies, evaluate the effect of different types of GBCAs on the gadolinium deposition in brain, introduce the possible entrance or clearance mechanism of the gadolinium and potential side effects of gadolinium deposition in brain, and put forward some suggestions for further research.

## The Physicochemical Properties of GBCAs

Gadolinium is a paramagnetic material which can shorten the T1 relaxation time of living tissues. Based on the type of ligand and charge, the commercially available GBCAs can be classified into 4 different types (Frenzel et al., 2008): linear ionic, linear non-ionic, macrocyclic ionic, and macrocyclic non-ionic. Macrocyclic GBCAs form a rigid cage including a preorganized cavity for Gd^3+^ ion, while linear ligands form more flexible cages that wrap around the Gd^3+^ ion and are not fully closed. The differences in thermodynamic and kinetic stability of those ligands may be caused by their different chemical structures, whereby the non-ionic linear chelates are the least stable and the ionic macrocyclic chelates are the most stable (Dekkers et al., 2017). **Table 1** gives the physicochemical properties of the commercially available GBCAs in current clinical practice.

**Table 1:** Biochemical properties of gadolinium-based contrast agents currently approved for clinical use.

Chemical structure	Trade name	Thermodynamic stability contrast	Conditional stability	Elimination pathway
**Linear**				
Nonionic				
Gadodiamide	Omniscan	16.8	14.9	Renal
Gadoversetamide	Optimark	16.6	15	Renal
Ionic				
Godopentetate dimeglumine	Magnevist	22.1	17.7	Renal
Gadobenate dimeglumine	Multihance	22.6	18.4	93%Renal; 3%Biliary
Gadoxeticacid disodium	Primovist	23.5	NA	50%Renal; 50%Biliary
Gadofosveset trisodium	Multihance	22	NA	91%Renal; 9%Biliary
**Macrocyclc**				
Nonionic				
Gadoteridol	Prohance	22.8	17.1	Renal
Gadobutrol	Gadavist	21.8	NA	Renal
Ionic				
Gadoterate meglumine	Dotarem	25.4	19	Renal

*NA indicates not applicable.*

## The Association Between High Signal Intensity on Unenhanced T1WI and Previous GBCAs Administrations In Human

**FIGURE 1:**
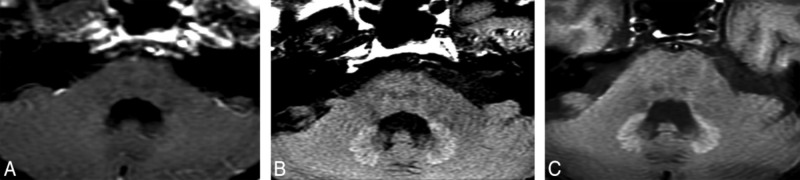
A patient with optical nerve pilocytic astrocytoma who treated with 5400-cGy radiation therapy and underwent 25 times CE-MRI scans show hyperintensity of the DN on unenhanced T1WI. DN initially appears as slightly hyperintense **(A)**. During the follow-up studies performed at 5 **(B)** and 8 **(C)** years after the initial hyperintense appearance of the DN, the signal intensity of the DN become more obvious. Image courtesy of Dr. Mehmet Emin Adin, Russell H. Morgan Department of Radiology, Division of Neuroradiology, Johns Hopkins Medical Institutions, Baltimore, MD. Images and text reproduced from reference 1 with written permission from AJNR.

T1-hyperintensity in the human brain is not a rare phenomenon. Many diseases such as multiple sclerosis (MS), Wilson disease, post-radiation therapy, hepatic encephalopathy (Roccatagliata et al., 2009; Kasahara et al., 2011, Kim et al., 2006; Rovira et al., 2008) etc., can lead to T1-hypeyintensity in deep gray matter. In the past 4 years, the correlation between previous GBCAs administrations and T1-hyperintensity in the deep brain has become a hot topic. Kanda et al. (2014) firstly described the T1-hypeyintensity in GP and DN may be caused by the previously repeated linear chelates GBCAs administrations. In this study, 19 adult patients with brain tumors previously receiving at least 6 doses of linear GBCAs (gadopentetate dimeglumine or gadodiamide) and 16 patients only receiving at least 6 unenhanced MRI without the GBCAs administrations were included. After calculating the mean signal intensity of the DN, GP, pons, and thalamus on unenhanced T1WI, T1-hyperintensity in deep gray matter nuclei only occurred in patients who previously exposed to GBCAs. The hyperintensity of DN and GP on unenhanced T1WI was associated with the number of previous GBCAs administrations independent on patients’ renal function. In the same year, Errante et al. (Errante et al., 2014) found T1 hyperintensity of the DN was common in patients with brain metastases who had undergone multiple gadolinium-enhanced brain MRI. In this study, 37 patients with brain metastases and 38 patients with MS underwent at least 2 consecutive CE-MRI examinations, and the results showed there were a linear relationship between T1 hyperintensity and the number of CE-MRI scans in patients both with brain metastases and MS. Since then, many studies demonstrated that repeated administrations of various types of linear GBCAs were associated with T1-hyperintensity in the brain (Weberling et al., 2015; Ramalho et al., 2016b; Kuno et al., 2017; Zhang et al., 2017). **Figure 1** shows a representative case with hyperintensity of the DN following repeated CE-MRI scans.

The same findings were also reported in pediatric patients. Roberts and Holden (2015) described a pediatric patient with repeated GBCAs administrations, progressive hyperintensity on unenhanced T1WI involving the DN and GP was observed. Similar phenomenon was also reported by others (Miller et al., 2015; Flood et al., 2016; Hu et al., 2016; Roberts et al., 2016a). **Table 2** presents the current evidences of gadolinium deposition in pediatrics and adult brain.

**Table 2:** Association between CNS structures hyperintensity and GBCAs administration in human.

Study	Groups	Contrast agent	MFS and sequence	Remarks
**Adult study**				
Kanda et al., 2014	19 patients underwent more than 6 CE-MRI examinations; 16 patients underwent more than 6 unenhanced examinations	Gadopentetate-dimeglumine Gadodiamide	1.5T	High SI in the DN and GP was associated with the number of previous CE-MRI examinations
Errante et al., 2014	38 patients with MS underwent more than 2 times CE-MRI scans; 37 patients with brain metastases underwent more than 2 CE-MRI scans	Gadodiamide	1.5T and 2-D TSE T1-weighted	Increase signal intensity on unenhanced T1WI has a linear relationship with the CE-MRI in patients with MS and BM
Weberling et al., 2015	50 patients underwent more than 5 CE-MRI	Gadobenate-dimeglumine	1.5T T1-weighted spin echo 3.0T fast low-angle shot	The SI ratio in the DN was increased after serial gadobenate dimeglumine administrations
Adin et al., 2015	184 patients treated with brain irradiation underwent 2677 MRI studies	Almost exclusively Gadopentetate-dimeglumine	1.5 or 3.0T MPRAGE, Spin-echo sequence and T1WI FLAIR	Repeated CE-MRI examinations likely results in persistent hyperintensity in the DN on unenhanced T1WI
Zhang et al., 2017	13 patients with more than 39 chelates GBCA administrations	Gadodiamide Gadopentetate-dimeglumine Gadobenate	1.5T or 3.0T 2D spin echo T1-weighted sequence 3D spoiled gradient-echo volume sequence	Increased SI on unenhanced T1WI was seen in the posterior thalamus, substantia nigra, red nucleus, cerebellar peduncle, colliculi, DN, and GP
Ramalho et al., 2016b	18 patients with previous gadodiamide and current gadobenate dimeglumine exposed; 44 patients with only gadobenate dimeglumine exposed	Gadodiamide Gadobenate-dimeglumine	1.5T fast spin echo T1-weighted images	Compared to those patients without previously gadodiamide-exposed, the prior gadodiamide-exposed groups show greater T1 SI changed
Kuno et al., 2017	9 patients received 1-8 times gadopentetate dimeglumine; 26 patients without prior GBCA exposure	Gadopentetate-dimeglumine	1.5T mixed fast spin echo pulse sequence	GBCA exposure patients show shorter T1 value compared with patients without prior GBCA exposure
**Pediatrics study**				
Miller et al., 2015	A pediatric patient who received 35 doses of linear GBCA during 12 years	Gadopentetate-dimeglumine	1.5T T1-weighted turbo-spin-echo	The DN, GP, and posterior thalamus showed visually evident increase in hyperintensity over the cause of repeated CE-MRI scans
Roberts and Holden, 2015	A 13 years old girl with follow-up CE-MRI scans	Gadopentetate-dimeglumine	1.5T or 3.0T	With the increasing use of GBCAs, hyperintensity was noted within both the DN and GP bilaterally
Roberts et al., 2016a	16 patients underwent more than 5 consecutive CE-MRI scans	Gadopentetate-dimeglumine	1.5T or 3.0T T1-weighted spin-echo Sequence	The number of prior GBCA doses is significantly correlated with progressive T1-weighted DN hyperintensity
Hu et al., 2016	21 patients received 5–37 times CE-MRI scans during their medical treatment; 21 controls of similar age without GBCA exposed	Gadopentetate-dimeglumine	1.5T T1 weighted 2-D fast spin echo	In all 21 patients with GBCA exposed, increased SI ratios were 18.6% ± 12.7% for the DN, and 12.4% ± 7.4% for the GP between the first and the most recent MRI scans
Flood et al., 2016	46 pediatrics patients underwent more than 3 times CE-MRI scans; 57 age-matched GBCA-naive control subjects	Gadopentetate-dimeglumine	1.5T T1-weighted spin-echo sequence T1-weighted 3-D MPRAGE sequence	SI in the pediatric brain increases on unenhanced T1-weighted MRI with repeated exposure to linear GBCA

*MFS, Magnetic Field Strength; SI, Signal intensities, DN, Dentate nucleus; GP, Globus pallidus; CE-MRI, Contrast enhanced magnetic resonance imaging; MS, Multiple sclerosis; BM, Brain metastases; FLAIR, Fluid attenuated inversion recovery; MPRAGE, magnetization prepared rapid acquisition of gradient echo sequence.*

## Histological Analysis Confirmed Gadolinium Deposition in Human Brain

The association between previous GBCAs administrations and T1 hyperintensity in DN and GP has been confirmed via human brain autopsy studies and animal studies. McDonald et al. (2015) compared the gadolinium contents of 23 (10 in contrast group and 13 in control group) deceased patients’ DN, pons, GP, and thalamus tissues by transmission electron microscopy, ICP-MS, and light microscopy. They found patients in the contrast group had 0.1–58.8 μg gadolinium per gram of tissue with a significant dose-dependent relationship, which was consistent with changes of signal intensity on unenhanced T1WI. It seemed the gadolinium could pass the BBB and deposit in the capillary endothelium and neural interstitium in the patients from contrast group who had relatively normal renal function when undergoing MR examination. Kanda et al. (2015a) confirmed the gadolinium accumulation in the brain after analyzing brain samples of five subjects who received GBCAs (gadopentetate dimeglumine, gadodiamide, gadoteridol) and five subjects with no history of GBCA administrations by ICP-MS. They found the inner segment of the frontal lobe cortex, cerebellar white matter, GP, and frontal lobe white matter had the detectable level of gadolinium accumulation, while the DN and GP had the highest level of gadolinium deposition. McDonald et al. (2017) compared postmortem neuronal tissue samples from five patients with 4–18 times CE-MRI examinations by using gadodiamide as contrast agent. They found gadodiamide-exposed patients had 0.1–19.4 μg of gadolinium per gram of tissue, and gadolinium was accumulated in the neuronal interstitium and capillary endothelium, even in the cell nucleus. All the aforementioned studies only detected the gadolinium deposition in patients with linear GBCAs administrations. One study by Murata et al. (2016) evaluated whether macrocyclic chelates GBCAs might deposit in human brain. In this study, tissue samples were acquired from 9 decedents undergoing autopsy who received CE-MRI examination using only single type of non-NSF related GBCAs, including gadoteridol (*n* = 5), gadobutrol (*n* = 2), gadobenate (*n* = 1), and gadoxetate (*n* = 1). They found gadolinium deposited in various brain areas including the caudate nucleus, putamen, cerebral white matter, and pons, with the highest levels in DN and GP for all patients. This finding indicated gadolinium deposition could be independent on gadolinium types. However, more evidences are needed to further confirm this finding via sensitive ICP-MS brain tissue analysis. **Table 3** presents the currently available reports of gadolinium deposition in brain with histological evidences. However, changes of T1 signal intensity are non-specific and can appear in several other pathological conditions such as calcium, manganese, iron, lipid, and other substances (Ginat and Meyers, 2012), further examinations and careful analysis are needed to verify what is responsible for the T1 signal intensity change.

**Table 3:** Autopsy studies in human or animal models.

	Group	Contrast agent	Detection methods	Remarks
**Human study**				
McDonald et al., 2015	13 patients with more than 4 GBCA administrations; 10 patients without GBCA exposed	Gadodiamide	ICP-MS; Transmission electron microscopy; Light microscopy	Gadolinium CNS structures deposition was associated with GBCA administrations and was independently with patients age, sex, renal function or interval between GBCAs exposed and death
Kanda et al., 2015a	5 patients received linear GBCAs before death; 5 patients with no history of GBCAs exposed before death	Gadopentetate-dimeglumine Gadodiamide Gadoteridol	ICP-MS	Gadolinium was deposited in the brain even in subjects without severe renal dysfunction, the highest accumulation area was the DN and GP
Murata et al., 2016	5 received gadoteridol; 2 received gadobutrol; 1 received gadobenate; 1 received gadoxetate; 9 patients without GBCAs exposed	Gadoteridol Gadobutrol Gadobenate Gadoxetate	ICP-MS	Gadolinium was found with all agents in all brain areas sampled with highest levels in GP and DN
Mcdonald et al., 2017	5 patients underwent 4-18 times CE-MRI examinations; 10 patients with no history of GBCAs exposed	Gadodiamide	ICP-MS; Transmission electron microscopy with energy-dispersive x-ray spectroscopy; Light microscopy	The patient DN, pons, GP and thalamus, contained 0.1–19.4 μg of gadolinium per gram of tissue in intracranial normality patients
**Animal models**				
Robert et al., 2015a	7 rats with gadodiamide-treated; 7 rats with gadoterate; meglumine-treated 7 rats with hyperosmolar saline-treated	Gadodiamide Gadoterate meglumine	ICP-MS	Repeated administration of the gadodiamide but not for the gadoterate meglumine were associated with T1-hyperintensity in the DN
Robert et al., 2015b	8 rats with gadobenate dimeglumine-exposed; 8 rats with gadopentetate dimeglumine-exposed; 8 rats with gadodiamide-exposed; 8 rats with gadoterate; meglumine-exposed 8 rats control group with saline injection	Gadobenate-dimeglumine Gadopentetate-dimeglumine Gadodiamide Gadoterate-dimeglumine	ICP-MS R1-mapping	Linear chelates GBCAs, gadodiamide, gadobenate dimeglumine, gadopentetate dimeglumine were associated with T1 hyperintensity in the DN along with gadolinium deposition in the cerebellum while gadoterate meglumine-exposed rats with no abnormal signal intensity observed
Lohrke et al., 2017	10 rats received gadodiamide; 10 rats received gadopentetate dimeglumine; 10 rats received gadobutrol; 10 rats received gadoteridol; 10 rats received saline as control group	Gadodiamide Gadopentetate-dimeglumine Gadobutrol Gadoteridol	ICP-MS; LA-ICP-MS; Scanning electron microscopy coupled to energy dispersive x-ray spectroscopy and transmission electron microscopy respectively	The administration of linear GBCAs was associated with significant high gadolinium concentration in the brain and skin compared to macrocyclic GBCA administration, however, no histopathological findings were detected in the rat’s brain.
Boyken et al., 2018	8 pigs received gadobutrol and gadopentetate dimeglumine 5 received gadobutrol only	Gadobutrol Gadopentetate-dimeglumine	ICP-MS	Repeated gadobutrol exposure is not associated with gadolinium deposition in healthy pigs’ brain, but additional a single dose gadopentetate dimeglumine is sufficient for gadolinium accumulation in the DN and GP

*GBCA, Gadolinium-based contrast agent; ICP-MS, Inductively coupled plasma mass spectroscopy; LA-ICP-MS, laser ablation coupled with ICP-MS; CNS, Central nervous system; DN, Dentate nucleus; GP, Globus pallidus.*

## The Effect of Gbcas Types on the Gadolinium Deposition

Although autopsy studies have shown all GBCAs can lead to gadolinium deposition in brain, most clinical studies indicated linear GBCAs had more detectable gadolinium deposition than macrocyclic GBCAs. Radbruch et al. (2015) compared signal intensity ratios of the GP and the DN to other structures on unenhanced T1WI in patients with linear chelates or macrocyclic chelates GBCAs exposure. Their study included two groups of 50 patients who received at least six consecutive MRI examinations exclusively using either a macrocyclic GBCA (gadoterate meglumine) or a linear GBCA (gadopentetate dimeglumine). They found signal intensity increase in the DN and GP on unenhanced T1WI was caused by serial application of the linear GBCAs rather than macrocyclic GBCAs. Kanda et al. (2015b) evaluated 73 patients who had previously exposed to GBCAs (23 patients received linear GBCAs, 36 patients received macrocyclic GBCAs, and 14 patients received both types of GBCAs) and 54 cases with no history of administrating any kind of gadolinium chelates. They found that T1-hyperintensity in the DN was related with previous use of linear GBCA (gadopentetate dimeglumine) rather than macrocyclic GBCA (gadoteridol). Gadoxetic acid, or gadoxetate disodium has been used as a liver-specific MRI contrast agent since its introduction in 2008, and there was no study showing possible brain gadolinium deposition after using gadoxetic acid. Ichikawa et al. (2017) compared 33 patients with more than 5 times of gadodiamide exposure (linear-nonionic) with 33 patients with more than 5 times of gadoxetic acid exposure (linear-ionic) and found T1-hyperintensity in the DN was related with previous gadodiamide administrations but not gadoxetic acid administrations. However, the administrated gadolinium dose in gadoxetic acid was only a quarter of that in gadodiamide, which might influence the results of this study.

Many animal studies also supported the viewpoint that linear GBCAs had more detectable gadolinium deposition in brain than macrocyclic GBCAs did (Jost et al., 2015; Robert et al., 2015a,b; Lohrke et al., 2017; Boyken et al., 2018). For example, Robert et al. (2015a) compared the signal intensity of DCN and the DN in healthy rats after exposing to some linear and macrocyclic agents. They divided 21 rats into 3 groups: gadodiamide-treated group, gadoterate meglumine-treated group and hyperosmolar saline group. This study showed repeated use of gadodiamide (linear agent) in healthy rats was related with persistent and progressive T1-hyperintensity in the DCN and gadolinium deposition in cerebellum, while in the gadoterate meglumine (macrocyclic agent) -treated group, no effect was observed. Boyken et al. (2018) collected the cerebellum, DCN, cerebral cortex, and pons of 15 pigs after repeated intravenous injections of GBCAs. Of the 15 pigs, 8 received up to 48 doses of gadobutrol and gadopentetate dimeglumine, 5 received up to 29 doses of gadobutrol only, and 2 had no GBCA administrations. After analyzing the gadolinium concentration in the aforementioned brain areas by ICP-MS, they found gadobutrol exposure was not associated with gadolinium deposition in pig brain while even a single additional administration of gadopentetate dimeglumine was sufficient for gadolinium accumulation in the DN and GP.

## Macrocyclic Chelates GBCAs and Gadolinium Deposition in Human Brain

Although many studies showed the linear GBCAs were associated with the gadolinium deposition in human brain, some studies indicated macrocyclic chelate GBCAs also lead to T1-hyperintensity in brain. Stojanov et al. (2016) revealed an obvious signal intensity increase in both GP and DN on unenhanced T1WI in patients with RRMS following multiple gadobutrol administrations. They divided 58 patients into 3 groups (27, 96–98, and 118 weeks) based on the intervals of contrast administrations. Patients receiving contrast agents with 27 weeks interval showed the largest increase in GP-to-thalamus signal intensity ratio, while a decrease in the GP-to-thalamus signal intensity ratio was found in patients receiving contrast agents with intervals of 96–98 weeks. However, this study did not consider other confounding factors such as other causes or disease process resulting in T1 hyperintensity, previous use of other contrast agents, and disease activity itself (Roccatagliata et al., 2009; Agris et al., 2016). Bjørnerud et al. (2017) reported two patients without any linear GBCA administrations before and received 37 and 44 doses of gadubutrol respectively, and they found visually appreciable enhancement of the DN on unenhanced T1WI, which showed a significant linear association with the number of macrocyclic GBCA injections. This finding should be further confirmed in larger and better controlled prospective studies. Based on current evidences, it suggests that both linear and macrocyclic GBCAs can cause gadolinium retention, despite the very different levels of total gadolinium exposure.

Similarly, the controversy about whether the macrocyclic chelates may induce hyperintensity in the specific brain structures in pediatrics also exists. Although many studies (Radbruch et al., 2017; Renz et al., 2017; Tibussek et al., 2017; Ryu et al., 2018) reported that only the linear chelates were associated with T1-hyperintensity in the brain while the macrocyclic chelates were not responsible for this change, some researchers also found that the administration of some macrocyclic chelates (gadoterate meglumine) was associated with T1 signal intensity changes in children (Mcr et al., 2017).

## Gadolinium Deposition Beyond The Brain

Gadolinium-based contrast agents can not only deposit in the brain, but also in the skin, bone, liver, and other organs. Gibby et al. (2004) found that gadodiamide or gadoteridol could deposit in the bones in patients undergoing total hip arthroplasty through inductivity coupled plasma atomic emission spectroscopy. Many other researchers (White et al., 2006; Darrah et al., 2009; Lord et al., 2017) confirmed this finding. There was a positive correlation between the dose of GBCAs administration and the gadolinium concentration measured in bone. It’s worth mentioning that macrocyclic chelates (gadobutrol) could accumulate in the bone and retain for 5 years after one injection (Lord et al., 2017), and autopsy study (Murata et al., 2016) demonstrated gadolinium level in the bone was 23 times higher than in the brain, which suggest that bone may act as a long-term storage site for gadolinium in the body. Roberts et al. (2016b) reported gadolinium accumulation in the skin of one patient with normal renal function after exposure to 61 cumulative doses of GBCAs. Maximova et al. (2016) reported liver gadolinium deposition occurred in pediatric patients with iron overload but normal hepatic and renal function following CE-MRI examinations.

Being similar as the results in humans, the rat models (McDonald et al., 2017) showed significantly elevated levels of elemental gadolinium in hepatic, splenic, and renal tissues following administration of high doses of GBCAs, although no tissue injury was observed in these organs.

## Potential Pathway of GBCAs Entering Into the Brain

There are two barrier systems in human brain, the blood-CSF and the BBB barrier. In normal conditions, GBCAs can not penetrate the intact BBB (Weinmann et al., 1984), while various illness conditions may affect BBB integrity and function and allow the GBCAs enter into the brain. Previous studies showed that gadolinium could deposit in the brain without intracranial abnormality (McDonald et al., 2017). Jost et al. (2015) evaluated the infiltration and distribution of 5 commercial GBCAs (gadopentetate dimeglumine, gadobenate dimeglumine, gadodiamide, gadoterate meglumine, gadobutrol) into the CSF in healthy rats. This study demonstrated the above-mentioned 3 linear chelates can increase and persist the DN to pons signal intensity ratios during up to 24 days observation. A CSF signal enhancement on postcontrast fluid-attenuated inversion recovery images (FLAIR) was found for all GBCAs independent of their chemical structure. Thus, the GBCAs passing from the blood into CSF might represent an initial pathway of GBCA infiltration into the brain. Nevertheless, to date, the underlying mechanism of gadolinium distribution into the DN and the GP remains unclear and needs be further studied.

## Clearance of Gadolinium from the Brain

Previous studies focused on the signal intensity changes on unenhanced T1WI MRI and the histological analysis on gadolinium deposition, however, few studies to date have been conducted to investigate whether those materials were cleared. The hyperintensity disappearance of the DN during follow-up was firstly described by Adin et al. (2015). After that, Smith et al. (2016) measured the gadolinium deposition level in healthy rats in 1 and 20 weeks after administrating linear chelates gadodiamide with up to 20 repeated doses. One week after dosing, gadolinium was detected in the brain at 0.00019% of the injected dose, and 20 weeks later, this diminished by approximately 50% (0.00011% of the injected dose). This study demonstrated partial clearance of the agents occurred over 20 weeks, however, it remains to be further confirmed in humans. Behzadi et al. (2018) conducted a retrospective study in 13 patients with previous more than 6 times gadopentetate dimeglumine (linear chelate GBCA) administrations then switching to gadobutrol (macrocyclic chelate GBCA) administrations. They found the developed DN–to-pons and DN–to–cerebellar peduncle signal intensity ratios decreased during mean follow-up time of 28 months after the last gadopentetate dimeglumine administration. The mechanism of the decreased signal intensity ratio was ascribed to the GBCAs washout hypotheses, chelators in the patient’s diet or medications, or the mixture of discussed mechanisms. However, the interaction between signal intensity and repeated gadobutrol administrations during follow-up can be further studied (Adin and Yousem, 2018). As for the washout hypothesis, some researchers (Smith et al., 2016) approved the findings while other studies (McDonald et al., 2015; Robert et al., 2015b) did not demonstrate. These inconsistent results indicated that if washout occurs, maybe it is in very low level because earlier observation demonstrated no washout while longer follow-up imaging showed washout sign (Ramalho and Ramalho, 2017).

## Potential Impacts of GBCAs Administrations

With repeated GBCAs administrations, gadolinium can deposit in brain and other organs even in patients with normal renal function. However, the clinical implication of the gadolinium deposition in the brain remains poorly understood.

Although some adverse effects of gadodiamide administration were reported, no strong evidences showed gadolinium deposition in the brain induced adverse clinical effects. Prince et al. (2011) reported gadodiamide administration caused spurious hypocalcemia, especially in patients with renal insufficiency and at doses of 0.2 mmol/kg or higher. Burke et al. (2016) did a survey about patients’ self-described toxicity related to GBCAs administrations. In this study, the most common symptoms were bone/joint pain and head/neck symptoms including vision, headache and hearing change. The study had a long list of limitations such as selection bias and validity problems, but constituted the first depiction of symptoms, which may be associated with gadolinium toxicity. One study by Bussi et al. (2017) evaluated the toxic effects of single and cumulative doses of gadobenate dimeglumine in neonatal and juvenile rats after receiving either saline or gadobenate dimeglumine at doses of 0.6, 1.25, or 2.5 mmol/kg. The authors reported no effects of gadobenate dimeglumine on cognitive function, behavior or any other parameters of rats, even at the highest administrated cumulative dose (15 mmol/kg). Thus, they concluded gadolinium in juvenile rat brain receiving single or cumulative gadobenate dimeglumine injection was minimal and non-impactful.

Gadolinium can deposit in the DN and GP and the potentially damaged GP may induce Parkinsonian symptoms. Welk et al. (2016) performed a population-based study to assess the relationship between parkinsonism and gadolinium exposure. In this study, 246 557 patients underwent at least one MRI examination during the study, there were 99 739 patients receiving at least one dose of gadolinium, and 2446 patients receiving 4 or more CE-MRI examinations. The results demonstrated the incident parkinsonism developed in 1.17% of gadolinium exposed patients and 1.16% of unexposed patients, and no significant association between parkinsonism and gadolinium exposure and parkinsonism was discovered. Perrotta et al. (2017) performed a retrospective study about the clinical cerebellar syndrome caused by gadoterate administration in ten patients who had previously received more than 20 doses of gadoterate. During 91-month follow-up, neither appearance of a rising cerebellar syndrome nor newly appeared symptoms or signs suggesting cerebellar toxicity were reported by the clinician.

## Potential Confounding Variables on Gadolinium Deposition in Brain

### Renal Function

Due to the majority of GBCAs excretion through the kidney, the researchers firstly concerned about the impact of renal function on the gadolinium deposition. Kanda et al. (2015a) analyzed brain tissues of 5 subjects and found out GBCAs accumulated in brain of subjects without severe renal dysfunction. Thus, even if the patient has normal renal function, GBCAs can still deposit in the human body. In patients with renal dysfunction, the condition of gadolinium accumulation in the body remains poorly understood. Kartamihardja et al. (2016) found renal dysfunction increased short-term gadolinium deposition in the bone, liver, skin, spleen, and kidney but it did not impact long-term gadolinium deposition. Rahatli et al. (2018) compared 13 patients on chronic hemodialysis who underwent 78 CE-MRI examinations with linear chelates GBCA (gadoversetamid) administrations with 13 patients with normal renal function, and they found the gadolinium accumulation rate in the brain after linear GBCA exposure may be affected by renal function. So, only in necessary conditions, GBCAs should be used and stabilized forms should be preferred.

## Patients’ Disease Condition

### Multiple Sclerosis

Multiple sclerosis patients need undergo brain MRI examinations both during periods of relapse and remission (every few months) to evaluate disease activity. The DN T1 hyperintensity was observed in patients with multiple sclerosis (Roccatagliata et al., 2009; Agris et al., 2016). This T1 hyperintensity was attributed to clinical disability, lesion load and brain atrophy (Rahatli et al., 2018), but not GBCAs administrations. Therefore, multiple sclerosis patients have been excluded from most investigations studying brain GBCAs deposition. However, some studies about the influence of different types of GBCAs administrations on gadolinium deposition were conducted in patients with MS and came to different results. In these studies, some researchers (Stojanov et al., 2016) reported DN-to-pons signal intensity ratios increased after repeated macrocyclic GBCAs administrations, while other researchers (Schlemm et al., 2016; Splendiani et al., 2017) concluded that multiple doses macrocyclic chelates administrations were not related to brain signal intensity changes in multiple sclerosis patients.

It appears disease spectrum has been associated with gadolinium deposition in human. One study (Tanaka et al., 2016) compared the gadolinium deposition difference between NMOSD patients and multiple sclerosis patients, and they showed patients with multiple sclerosis were prone to gadolinium accumulation while NMOSD patients had less tendency. The authors believed the differences in disease pathology and structure of gadolinium might impact gadolinium deposition.

## Brain Irradiation

Radiation-related brain injury may lead to calcifications, which can present as high signal intensity on the T1WI (Suzuki et al., 2000). Therefore, patients who have experienced brain irradiation are usually excluded from the studies investigating the relation of hyperintensity signal changes with previous administration of gadolinium. Tamrazi et al. (2017) observed 144 pediatric patients with CE-MRI examinations, including 55 patients with primary brain tumors and whole-brain irradiation, 19 with primary brain tumors and chemotherapy only, 52 with primary brain tumors without any treatments, and 18 with neuroblastoma without brain metastatic disease. This study demonstrated that at fewer numbers of GBCAs administration (≤10 times), whole-brain irradiation seemed to play a greater role in increasing the T1 signal intensity on unenhanced T1WI than administered GBCAs, while at a higher number of GBCAs administration (≥20 times), GBCAs seemed to contribute more to the increased signal intensity.

## Magnetic Field Strength, MRI Sequence and Interval Time Between Examinations

Although many gadolinium deposition studies have been published, one major limitation of these studies is the variability of the MR imaging protocols. One study showed magnetic field strength might affect the T1 signal intensity, however, magnetic field strength did not change the reader’s final diagnosis in any of the cases (Adin et al., 2015). Pulse sequence may directly or indirectly affect the detectability of T1 hyperintensity in the brain. Ramalho et al. (2016a) evaluated the T1 hyperintensity using spin echo (SE) sequence and magnetization prepared rapid gradient echo (MPRAGE) sequence and the result showed the DN/MCP signal intensity ratios were significantly higher when using SE than MPRAGE sequence. Thus, the two sequences should not be used interchangeably, and the baseline and final signal intensity ratios should be assessed by the same sequence. They recommended MPRAGE sequence as a simple screening tool in clinical practice for assessing patients with multiple GBCA exposure because of its intrinsic higher gray–white matter contrast. The interval time between baseline and final MRI examination (range: 96–1905 days) did not influence the final observation between the two sequences.

## Regulatory Statements

Since the first report of gadolinium deposition in brain, many official regulatory statements have been published to warn the safe use of GBCAs. In 2017, the International Society of Magnetic Resonance in Medicine (ISMRM) Safety Committee claimed that no direct evidences from human beings or animal studies demonstrated any harmful effects related to the gadolinium deposition in the brain. However, several recommendations for the use of GBCAs in clinics and research were provided by ISMRM. The main recommendations are as follows: (1) The ISMRM urges caution in the use of gadolinium-based contrast agents, and GBCAs should not be used when not necessary; (2) The clinical indication and pertinent information about GBCAs administrations should be documented in the patient’s medical record; (3) When choosing a gadolinium-based contrast agent, many factors should be considered, including pharmacokinetics, efficacy, relaxivity, patient age, potential side effects (like allergic reactions), probability of the need for repeated examinations, and cost. Institutions should evaluate these factors and consider that some agents might be with a greater propensity for deposition than others (Gulani et al., 2017).

In December 19, 2017, the US Food and Drug Administration (FDA) reviewed available data about the gadolinium retention from GBCAs as part of its role in monitoring the post-market safety of drugs. They recommended that health care professionals should evaluate the retention characteristics of each agent when using a GBCA in patients with higher risk, such as those who may require repeated GBCA MRI scans to monitor a chronic condition. The FDA also stated as “a class-wide warning about gadolinium retention in the labeling of GBCAs and a new medication guide that should be presented to patients in advance of receiving a GBCA” (U.S. Food and Drug Administration, 2017).

The Pharmacovigilance Risk Assessment Committee (PRAC) of the European Medicines Agency (EMA) confirmed (European Medicines Agency, 2017) restrictions on using linear gadolinium agents from July 7, 2017, the PRAC recommended that the intravenous linear agents gadobenic acid and gadoxetic acid should only be used for liver scans when they meet an important diagnostic need. Additionally, gadopentetic acid should only be applied for joint scans because the gadolinium concentration in the formulation used for joint injections is very low. All other intravenous linear agents (gadoversetamide adopentetic acid, and gadodiamide) should be suspended in line with the PRAC’s March 2017 recommendation. Another kind of gadolinium agent known as macrocyclic agents (gadoteric acid, gadobutrol, and gadoteridol) is more stable and has a lower tendency to release gadolinium than linear agents. These can continue to be applied in their current indications but at the lowest doses that enhance images sufficiently and only when unenhanced body scans are not appropriate.

## Future Perspectives

Although rapidly increasing number of published articles, the knowledge on gadolinium deposition in the brain and its clinical significance is still insufficient. And there exist many unsolved problems and further studies are needed. Firstly, whether the deposited gadolinium in the brain may result in any clinical consequences unreported during periods of study observation. Large prospective randomized controlled trial should be performed to clarify this important issue. Secondly, the mechanism of GBCA deposition in the brain remains unknown, and several related problems are still unsolved, including the initial pathway of gadolinium entering into the brain, the potential saturation and washout effects, and the exact molecular structure of residual gadolinium. Thirdly, the mechanism of disease itself affecting the gadolinium deposition in the brain is still unknown. Are some groups of patients prone to gadolinium deposition in brain? Future studies should focus on these unsolved problems and give valid evidences to the public.

## Conclusion

Recently studies have confirmed gadolinium accumulation in human brain following repeated gadolinium based contrast agent administrations, regardless of an intact BBB or normal renal function. Linear chelates GBCAs can result in more gadolinium deposition than macrocyclic chelates GBCAs. However, the impact of the retained gadolinium in the brain remains unknown, which needs large prospective studies to clarify in future. It is recommended to take caution when using macrocyclic chelates GBCAs and keep as low doses as possible for reducing gadolinium accumulation in brain.

## Author Contributions

All authors listed have made a substantial, direct and intellectual contribution to the work, and approved it for publication.

## Conflict of Interest Statement

The authors declare that the research was conducted in the absence of any commercial or financial relationships that could be construed as a potential conflict of interest.

## Abbreviations

BBB: blood brain barrier
CE-MRI: Contrast enhanced magnetic resonance imaging
CSF: cerebrospinal fluid
DCN: deep cerebellar nuclei
DN: dentate nucleus
GBCAs: Gadolinium-based contrast agents
GP: globus pallidus
ICP-MS: inductively coupled plasma mass spectroscopy
NMOSD: neuromyelitis optica spectrum disorder
NSF: nephrogenic systemic fibrosis
RRMS: relapsing-remitting multiple sclerosis, T1WI, T1-weighted imaging.
